# Boston College daily sleep and well-being survey data during early phase of the COVID-19 pandemic

**DOI:** 10.1038/s41597-021-00886-y

**Published:** 2021-04-16

**Authors:** Tony J. Cunningham, Eric C. Fields, Elizabeth A. Kensinger

**Affiliations:** 1 Department of Psychiatry, Harvard Medical School, Boston, MA 02215 USA; 2 Department of Psychiatry, Beth Israel Deaconess Medical Center, 330 Brookline Ave, Boston, MA 02215 USA; 3 Department of Psychology and Neuroscience, 275 Beacon St, Chestnut Hill, MA 02467 USA; 4 Department of Psychology, Brandeis University, 415 South Street, Waltham, MA 02453 USA

**Keywords:** Psychology, Risk factors, Society

## Abstract

While there was a necessary initial focus on physical health consequences of the COVID-19 pandemic, it is becoming increasingly clear that many have experienced significant social and mental health repercussions as well. It is important to understand the effects of the pandemic on well-being, both as the world continues to recover from the lasting impact of COVID-19 and in the eventual case of future pandemics. On March 20, 2020, we launched an online daily survey study tracking participants’ sleep and mental well-being. Repeated reports of sleep and mental health metrics were collected from participants ages 18–90 during the initial wave of the pandemic (March 20 – June 23, 2020). Given both the comprehensive nature and early start of this assessment, open access to this dataset will allow researchers to answer a range of questions regarding the psychiatric impact of the COVID-19 pandemic and the fallout left in its wake.

## Abstract (summary)

**Table:** 

Measurement(s)	Emotional Well-being • sleep quality • worry measurment • social behavior • Stress • self rated health • Alcohol Consumption • Affective Symptoms • chronotype measurement • Anxiety • Personality Trait • Demographic Data • empathy measurement • COVID-19 Related Memory Questions • self-regulation • Emotion • behavioural disinhibition measurement • response to uncertainty
Technology Type(s)	Survey • Pittsburgh Sleep Quality Index • Generalized Anxiety Disorder - 7 Questionnaire • Perceived Stress Scale • Patient Health Questionnaire - 9 Item • Positive and Negative Affect Schedule • Ultra Short Munich Chronotype Questionnaire • Cognitive emotion regulation questionnaire (CERQ) • Liebowitz Social Anxiety Scale (LSAS) • Self-Report of Personality • Reduced Morningness Eveningness Questionnaire (MEQ) • Toronto Empathy Questionnaire • Brief Self-Control Scale • Emotion Regulation Questionnaire (ERQ) • Short Impulsive Behavior Scale • Intolerance of Uncertainty Scale
Sample Characteristic - Organism	Homo sapiens
Sample Characteristic - Environment	first-order administrative region • human dwelling
Sample Characteristic - Location	United States of America • Global

Machine-accessible metadata file describing the reported data: 10.6084/m9.figshare.14069348

## Background & Summary

The outbreak of COVID-19 and the societal responses taken to combat its spread have had far reaching consequences, providing a unique opportunity to examine how large groups of individuals fare when exposed to chronic stress and uncertainty. The present study was designed to better understand the repercussions of the COVID-19 pandemic and the subsequent response measures associated with it (e.g. social distancing and “lockdowns”) on mental health and sleep patterns. In addition to the daily surveys, we collected comprehensive demographics and extensive assessments of factors related to well-being (e.g. prosocial behavior, emotion regulation strategies, tolerance of uncertainty, personality traits). The extent of the information collected on this large sample of global participants allows for investigation of a number of potential avenues of future investigation, such as individual differences in feelings of social isolation and exercise during the pandemic and how demographic variables such as age^1^ or minority status^2^ affect mental-health outcomes.

It is well documented that mental wellbeing suffers when under chronic stress, and particularly when individuals do not feel able to control the source of the stress^3^. It is also apparent that sleep can be impaired when under stress, and that changes in sleep can have negative consequences on emotions and mental wellbeing^4,5^. However, other evidence makes it clear that there are situational factors that can alter the resilience shown under stress. For instance, an extensive literature has revealed the importance of social connectedness for mental wellbeing (reviewed by^6–8^) and has shown that there are not only physical benefits to staying physically active but also mental health benefits^9,10^. Therefore we included a number of questions about these situational factors that warrant further exploration.

At the time that this study was designed, in March 2020, it was becoming apparent that the spread of COVID-19 was not going to be easily contained. There had been widespread focus on the physical-health repercussions of the pandemic but relatively less discussion of the mental-health repercussions of the chronic stress associated with the pandemic and the societal responses being taken. On March 20, 2020–a day after the first US state-wide “stay-at-home” order was issued in California–we launched an online survey study meant to capture the impact of the first wave of the pandemic on individuals’ mental wellbeing and sleep patterns. As made clear in a recent meta-analysis, the COVID-19 pandemic^11^ has led to a high prevalence of sleep disruptions, affecting approximately 40% of people from general and healthcare populations, suggesting the importance of the topic examined in our survey study.

The study was designed to be comprehensive and long-term in nature, asking participants to fill out daily sleep reports and assessments of their mental wellbeing. As the weeks passed, the benefits of this longitudinal design came into focus. The early days and weeks provide insight into how individuals fared as they initially adapted to stay-home recommendations, school closures, and changes to job structures. As time went on, the data provide insight into how individuals cope in the midst of a continuing public health crisis that also has had dire economic consequences^12^. Despite the ongoing challenges that individuals needed to confront, completed analyses have revealed an improvement in participants’ reported wellbeing over time: Individuals reported more stress, worry, and negative affect in the March and early April than they did in later April and May^1,2^. As made apparent through these analyses, the primary benefit our frequent and long-term assessment is that it permits a much more fine-grained analysis of changes in sleep and mental health as the days passed during the initial spread of the pandemic, as opposed to collecting snapshots of functioning at one or two time points.

At the time of this submission, the dataset includes a sample of 1,518 participants and 37,882 survey responses. Data has been collected from adults ages 18 to 90 and with an extensive demographic section covering location information (country and, in the case of US, state), employment status, COVID-risk level, race, ethnicity, and sexual orientation and gender identity. As such, these data will enable researchers to explore how the current pandemic experience is differently impacting people by age, economic impact, minority status, and risk-status. Additionally, we are planning a series of follow up assessments to add additional longitudinal information to this already rich dataset, which will also be made available on Open Science Framework (OSF).

## Methods

### Participants

Online recruitment for this dataset began on March 20, 2020 and ended August 5, 2020. In response to recruitment during this time frame, N = 1,899 individual participants completed the online informed consent and were enrolled in the study. Of this initial recruitment, N = 1,518 (age range: 18–90 years old; M = 35.2, SD = 15.1) completed the initial demographic survey, which was required before daily data collection began. All English-speaking adults 18+ were eligible for the study, regardless of pre-existing medical or mental health conditions. Demographics of the entire sample can be found in Online-only Table 1. For completion of daily surveys and one-time assessments described below, participants received entries into raffle drawings for gift cards as compensation. The Institutional Review Board at Boston College approved all consent and assessment procedures under IRB Protocol Number 20.212.01.

### Recruitment methods

Participants were recruited primarily via social-media postings (Facebook, Twitter, Reddit), direct emails to individuals who had expressed an interest in being contacted about research studies, and emails to listservs with members interested in relevant topics (e.g., scientific societies). The timing of the surveys was aligned with the first wave of the COVID-19 pandemic in North America, but attempts were made to encourage a more international sample by using social media outlets and listservs with international reach. However, surveys were only available in English and thus only English-speaking individuals could participate, limiting the ability to gain a representative international sample.

### Assessment materials and design

After consenting to the study, participants were assigned a unique and de-identified Participant ID. Participants were asked to enter the ID at the beginning of each assessment they completed for the duration of the study, and to protect the confidentiality of the participants these IDs were the only means of linking their data over time and across assessments. Further, to ensure anonymization, the Participant IDs have been replaced with numeric placeholders in the publicly available datasets. Study data were collected and managed using REDCap^13,14^ electronic data capture tools hosted at Boston College. REDCap is a secure, web-based software platform designed to support data capture for research studies. Along with their Participant ID, participants received a link to our initial demographic survey. After completion of the demographic survey, participants were placed in the pipeline to receive information on the rest of the assessments as described below for the duration of the study or until they requested to withdraw (<5%). PDF copies of all survey questions are available with the data on the OSF study page: 10.17605/OSF.IO/GPXWA.

#### Demographic survey

Following the consent protocol, participants received an email with their Participant ID and a link to the initial demographic survey. Participants were required to complete this demographic survey prior to receiving information on any further assessments. Participants were asked to report age, race and ethnicity, natal sex, gender identity, sexual orientation, socioeconomic and military status, education, marital status, number of dependents, and previous diagnoses of serious medical and mental health conditions. Further demographic data was collected in the *Round 3* one-time assessment (see below).

#### Daily survey

After completion of the initial Demographic Survey, participants were enrolled to begin receiving our daily survey assessment. To reduce participant burden, two versions of our daily survey were utilized during the assessment period: the *Short Version* and the *Full Version*. To establish a baseline of all metrics included in the daily surveys, participants received the Full Survey for at least three days following completion of the demographic survey. The Full Survey was then sent to all participants on randomly selected days 2 days/week, with the Short Survey sent the remaining 5 days/week. To enhance the quality of the data reported, participants were instructed not to try to make up surveys on days that they missed. The Short and Full Version of the daily surveys are described in detail below.

The *Short Version* of our daily survey included several questions relevant to the duration and quality of sleep, including bedtime, sleep attempt time, sleep latency, time spent awake after sleep onset, morning wake time, and the time participants got out of bed. We also collected daily dream reports, descriptions of activity and exercise, time spent virtually socializing, alcohol consumption, quarantine status, COVID-19 symptoms and diagnosis, and their subjective experience of overall stress. All questions within the Short Version of the survey were optional and participants were asked to respond to any that they were able to given their time and energy that day.

The *Full Version* of the survey included all questions from the Short Version, as well as questions related to their experience of worry on factors related to COVID-19 (*i.e*., individual health, health of family, friends, and community, public health, and financial impact), perception of social isolation, current mood using the Positive and Negative Affect Schedule (PANAS)^15^, and symptoms of depression using a modified version of the Patient Health Questionnaire-9 (PHQ-9)^16^ that omitted the question assessing suicidality. Most questions within the Full Version were required in order to be submitted, but participation was optional each day it was received.

Participants received either the Short or Full Version of the daily survey at 08:00 in their local time zone every day of the assessment period from March 21, 2020 - May 20, 2020. After May 20th, we discontinued the Short Version of the survey, but continued to send the Full Version of the survey 2 days/week from May 21, 2020 - June 23, 2020. Participants that enrolled in the study after June 23, 2020 only received the Full Version of the survey for the initial three days following completion of the demographic survey.

#### Round 1 Assessment

The Round 1 one-time assessment was launched on May 19, 2020. Initial invitations and reminders to complete the survey were sent via REDCap and email. The Round 1 assessment included the following previously validated measures: Pittsburgh Sleep Quality Index (PSQI)^17^, pre- and post-COVID assessment of the ultra-short Munich Chronotype Questionnaire^18^, Generalized Anxiety Disorder (GAD-7) Scale^19^, Cognitive emotion regulation questionnaire (short version)^20^, pre- and post-COVID assessment of the Liebowitz Social Anxiety Scale^21^, and a Big 5 Inventory-2 (Short Form)0^22^.

#### Round 2 Assessment

The Round 2 one-time assessment was launched on June 16, 2020. Initial invitations and reminders to complete the survey were sent via REDCap and email. The Round 2 assessment included the following measures: Insomnia Severity Index (ISI)^23^, Reduced Morningness Eveningness Questionnaire (RMEQ)^24^, Perceived Stress Scale^25^, Toronto Empathy Questionnaire^26^, and a series of questions specifically tailored for events surrounding the COVID19 pandemic. As part of these questions, we asked participants to reflect on their experience and memory of the onset of the pandemic from March - June, 2020 and to recount specific memories from this time-period and to report their general experience of how positive or negative they remember this period of time being. We also ask them to discuss how well their experience matched their initial predictions about the spread of the pandemic and assessed their future predictions for the success of an eventual reintegration process.

#### Round 3 Assessment

The Round 3 one-time assessment was launched on June 29, 2020. Initial invitations and reminders to complete the survey were sent via email only. The Round 3 assessment included the following measures: Short Urgency-Premeditation-Perseverance-Sensation Seeking-Positive Urgency (UPPS-P) Impulsive Behavior Scale^27^, Intolerance of Uncertainty Scale^28^, Emotion Regulation Questionnaire^29^, Brief Self-Control Scale^30^, and an Exit Survey collecting additional important demographic information that became apparent over the course of the assessment period and their experience as participants in the study. This included a more detailed assessment of pre-existing and current medical and mental health information, COVID-19 high risk factors, purchasing and use of personal protection equipment (PPE), information on essential workers and healthcare professionals, additional economic impacts of COVID19, implementation of stay-at-home orders or other government-ordered measures initiated in their area, and additional assessment of experience with COVID-related dreaming.

**Fig. 1:**
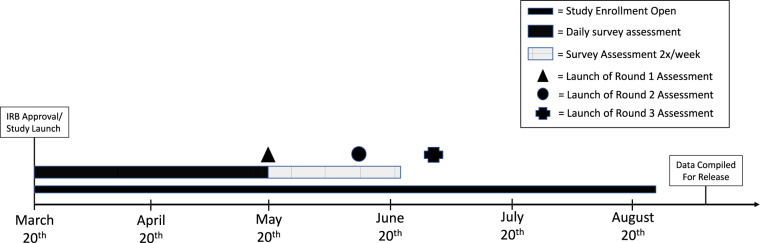
Schematic of study timeline. IRB approval and study launch occurred on March 20, 2020. Initial enrollment in the study was open until August 5, 2020. Daily survey assessments (a mix of the Full and Short Versions) took place from March 21, 2020 - May 20, 2020. The Full Version assessments then continued 2 days/week from May 21, 2020 - June 23, 2020. The one-time assessments were then released on the following dates: Round 1 – May 19, 2020; Round 2 - June 16, 2020; Round 3 – June 29, 2020.

All participants had the opportunity to complete the one-time assessments from their initial launch date until August 26th, 2020. Approximately 55% of the participants completed the first one-time assessment, and approximately 42% of participants completed the Round 2 and Round 3 assessments. Of the total sample, 37.2% (n = 564) completed at least one daily survey and all three rounds of the one-time assessments. The full timeline of the study can be found in Fig. 1.

## Data Records

All five datasets (demographics, daily survey, and Round 1–3 data) have been anonymised and both the raw and cleaned data are available in CSV (non-proprietary) formats on the Open Science Framework (OSF) platform^31^. We have also included PDF files of the questionnaires and README files in DOCX and PDF format that include variable descriptions and explanation of all data processing done in cleaned versions of the data sets. To assist with hypothesis generation that may be relevant to the spread of COVID-19 in different areas, we have also included an XLSX file with all participant locations that includes dates of first infection, peak infection, peak death rate, dates of lockdowns, and descriptions of lockdowns. All of these materials are available at the following link: https://doi.org/10.17605/OSF.IO/GPXWA.

## Technical Validation

A Python script was written to confirm that all variables were within expected ranges. Most variables were Likert-type scales that could only take on limited values (e.g., the integers 1 through 7). For these, we confirmed that all responses were in this set of possible values. For open-ended numeric responses (e.g., “How many people do you live with?”) we confirmed that all responses were numeric. Impossible values were replaced with missing values (e.g., a person who responded that they worked 8 days per week on average or someone who reported drinking over 12,000 alcoholic beverages on a given day), but merely implausible values (e.g., reported sleep time as 22.25 hours) were retained. It is therefore left to researchers using the dataset to determine a plan for dealing with outliers in these variables. For other open-ended responses, obvious mistakes were corrected (e.g., misspelling of a country name) but were otherwise left as is. Details of the data cleaning procedure are available in the README files provided with the data. The original, uncleaned data is also provided.

Descriptive statistics of all calculated variables were checked to make sure that results were within the expected range. The quality control script, which checks for proper formatting of all variables, reports percentages of unlikely values, and reports the number of missing values for each variable, is shared with the data. Online-only Table 2 contains output from a sample of variables/scales from each phase of the study design as a demonstration of this quality control check.

## Usage Notes

As noted, the data files on the OSF platform are accompanied by README files in DOCX and PDF format that include descriptions of variables within each dataset and explanations of all data processing that has been done in the processed versions of the data sets. The datasets include both responses to every individual question that was de-identifiable and calculated scale scores in the case of validated measures or composite metrics that we believe may be of interest to researchers. Descriptions of calculated scores can also be found in the README files.

### Limitations of the data

Users should keep in mind limitations about the sample, e.g., primarily White participants, skewed toward participants who are female, well-educated, and from the United States (with further skew toward Massachusetts residents) and limitations related to potential biases in attrition over time as well as biases in the days on which daily surveys may have been completed (e.g., participants were encouraged to attend first to their health and thus would likely have skipped surveys on days when they were not feeling well). Users of this dataset should also be aware that the measures rely on self-report, and often on subjective assessments, without any attempts to externally validate the accuracy of those reports.

Another important consideration of this dataset is that the completion of each daily survey and each one-time assessment was optional. As such, the number of entries per participant varies, and because some questions were optional, the sample size additionally varies across metrics. The number of people that have completed each survey and each survey element can be easily determined using the participant ID code provided. Prior to use, data users should be sure to set restrictions on the quantity and timing of the assessments as needed (e.g. minimum number of daily surveys completed, order and timing of completion of one-time assessments, etc.) or use statistical analyses that allow for this type of variability.

Finally, as discussed above in *Technical Validation*, a small proportion of free response questions contain some potentially implausible responses. This was most apparent in questions regarding the timing of sleep and wake as it both requires free response and REDCap can only verify a standard time entry in 24-hour format, which a minority of participants struggled to remember to do. Further, there are a variety of ways the participants could make errors (e.g. incorrect time in bed, incorrect rise time, using 12-hour instead of 24-hour time format, etc.) and we did not determine a way to safely and confidently catch all errors without making potentially incorrect assumptions or potentially losing good data along with bad. Importantly, concerns of implausible responses even within the sleep data affect only a small percentage of responses (e.g. 93% of ‘total sleep time’ responses between 4–10 hours, 2.6% of total sleep time responses <2 hours or >12 hours). As such, the goal of our initial data cleaning was to take a conservative approach: instead of trying to correct problems by making additional, potentially incorrect assumptions, we took responses at face value as much as possible. When results were impossible or ambiguous (but not simply unlikely), we then replaced the value with a missing value in the clean version of the data set, rather than trying to guess at the correct response. This still leaves a number of known issues in a small percentage of entries. We extensively describe the cleaning process, known issues, and make recommendations for investigators interested in utilizing the sleep data in the SLEEP_DATA_README files. Additionally, all original responses can be found in the data files labeled as “raw”.

## Data Availability

All code for formatting, cleaning, and quality assurance was written in Python (python.org) with use of the NumPy (numpy.org) and Pandas (pandas.pydata.org) libraries. This code is available on the studies OSF page, along with the code used to produce Online-only Tables 1 and 2 All code is released under a free and open source license (BSD three-clause): 10.17605/OSF.IO/GPXWA.

## Online-only Tables

**Online-only Table 1:** Demographics of participants that completed each phase of the study.

	All	Daily	R1	R2	R3
**N**	1518	1380	839	641	652
**Age**					
mean	35.22	35.39	37.76	39.22	38.83
std	15.09	15.14	16.55	17.43	17.16
min	18	18	18	18	18
25%	25	25	26	26	26
50%	30	30	31	33	32
75%	40	40	46	50	48.25
max	90	90	90	90	90
**Residence**					
USA	81.8%	81.9%	82.4%	81.7%	82.8%
Canada	3.0%	3.2%	3.7%	4.2%	4.0%
Australia	2.0%	2.0%	2.5%	3.0%	2.9%
United Kindgdom	1.3%	1.4%	1.0%	1.1%	0.9%
India	1.1%	0.9%	1.1%	0.8%	0.6%
other	10.7%	10.7%	9.4%	9.2%	8.7%
**Ethnicity**					
Hispanic	6.8%	6.3%	5.5%	4.7%	4.9%
Not Hispanic	91.6%	92.0%	92.5%	93.6%	93.4%
Prefer not to say (ethnicity)	1.7%	1.7%	2.0%	1.7%	1.7%
**Race**					
African American	1.5%	1.7%	1.7%	1.2%	1.5%
Asian	10.3%	9.9%	8.7%	8.1%	8.1%
White	76.9%	77.6%	79.7%	82.1%	81.1%
Hispanic/Latinx	3.0%	2.8%	2.4%	2.2%	2.5%
Native Hawaiian or Other Pacific Islander	0.1%	0.1%	0.1%	0.2%	0.2%
American Indian/ Alaska Native	0.1%	0.1%	0.1%	0.2%	0.2%
More than one race/Prefer to self-describe	7.0%	6.7%	6.1%	5.5%	5.8%
Unknown	0.3%	0.4%	0.5%	0.2%	0.2%
Prefer not to say (race)	0.7%	0.8%	0.7%	0.5%	0.5%
Gender					
female	78.2%	78.7%	82.0%	82.2%	82.5%
male	19.5%	19.1%	16.6%	16.4%	16.1%
non-binary/third gender	2.0%	1.8%	0.8%	0.8%	0.8%
prefer to self-describe	0.3%	0.3%	0.5%	0.5%	0.5%
prefer not to say	0.1%	0.1%	0.1%	0.2%	0.2%
**Biological Sex**					
female	80.1%	80.6%	82.8%	83.3%	83.4%
male	19.9%	19.4%	17.2%	16.7%	16.6%
**Gender Identity**					
cisgender	97.9%	98.3%	98.9%	98.9%	99.1%
transgender	1.4%	1.2%	0.7%	0.5%	0.3%
prefer not to say	0.7%	0.6%	0.4%	0.6%	0.6%
**Sexual Orientation**					
straight/heterosexual	79.2%	79.6%	82.1%	82.3%	81.8%
bisexual	13.6%	13.3%	12.4%	12.4%	12.6%
gay/lesbian	3.8%	3.9%	3.2%	3.3%	3.1%
prefer to self-describe	1.9%	2.0%	1.3%	1.3%	1.7%
prefer not to say	1.5%	1.3%	1.0%	0.8%	0.8%
**Education**					
some high school	1.1%	0.9%	0.6%	0.6%	0.3%
high school diploma or GED	3.0%	3.0%	2.3%	2.7%	2.5%
some college	16.7%	15.5%	13.9%	12.5%	13.0%
bachelor’s degree	27.9%	27.9%	26.1%	26.7%	27.5%
some post-bachelor	9.7%	10.2%	9.8%	10.0%	10.6%
graduate, medical, or professional degree	41.6%	42.5%	47.3%	47.6%	46.2%
**Relationship Status**					
single	34.4%	34.3%	30.9%	29.5%	30.5%
in a relationship	27.5%	27.6%	25.1%	24.3%	24.1%
married	31.6%	31.9%	37.3%	38.7%	38.0%
separated/divorced	4.9%	4.4%	4.5%	5.0%	4.9%
widowed	1.7%	1.7%	2.1%	2.5%	2.5%
**Serious medical problems?**					
yes	8.4%	8.0%	8.7%	8.1%	7.7%
no	91.6%	92.0%	91.3%	91.9%	92.3%
**Income**					
$0–25,000	11.3%	10.4%	8.7%	8.1%	8.1%
$25,001–50,000	15.3%	14.7%	14.9%	15.4%	15.5%
$50,001–75,000	17.7%	18.2%	18.4%	17.6%	17.2%
$75,001–100,000	16.1%	16.2%	16.9%	17.9%	17.5%
$100,001–150,000	18.3%	18.6%	18.6%	19.3%	19.8%
$150,001–250,000	13.1%	13.3%	13.3%	11.9%	11.8%
$250,000+	8.2%	8.6%	9.2%	9.7%	10.1%
**Are you a full time student?**					
yes	25.8%	25.7%	23.2%	22.9%	22.5%
no	74.2%	74.3%	76.8%	77.1%	77.5%
**Are you currently employed?**					
yes	77.4%	78.6%	75.8%	73.5%	74.9%
no	22.6%	21.4%	24.2%	26.5%	25.1%

All = All participants that enrolled in the study and completed the initial demographic survey; Daily = participants that successfully completed at least one daily survey; R1 = participants that completed the Round 1 one-time assessment; R2 = participants that completed the Round 2 one-time assessment; R3 = participants that completed the Round 3 one-time assessment.

**Online-only Table 2:** Technical validation of a subset of scales/variables from the complete dataset.

	M (SD)	Min	1st Quartile	Median	3rd Quartile	Max	Scale min	Scale Max	Assessment
# daily responses	23.35 (19.48)	1	6	19	38	71	—	—	daily
Stress	4.12 (1.53)	1	3	4	5	7	1	7	daily
Social isolation	4.24 (1.60)	1	3	4	6	7	1	7	daily
Worry Composite	18.56 (6.14)	5	14	18	23	35	5	35	daily
PANAS Positive Affect	22.72 (8.88)	10	16	21	28	50	5	50	daily
PANAS Negative Affect	16.25 (6.41)	10	11	14	19	50	10	50	daily
PHQ-9 (modified)	5.97 (4.83)	0	2	5	9	24	0	24	daily
Total sleep time	7.52 (1.54)	0.00	6.70	7.58	8.42	22.25	0.00	24.00	daily
Time in bed	9.13 (1.67)	0.02	8.10	9.00	10.00	23.85	0.00	24.00	daily
Sleep efficiency	0.83 (0.12)	0.00	0.77	0.85	0.91	1.00	0.00	1.00	daily
Pittsburgh Sleep Quality Index	6.20 (3.25)	0	4	6	8	18	0	21	R1
GAD-7 Score	6.37 (5.05)	0	2	5	9	21	0	21	R1
Insomnia Severity Index	8.34 (6.08)	0	3	7	12	28	0	28	R2
Perceived Stress Scale	19.07 (7.19)	0	14	19	24	40	0	40	R2
ERQ Cognitive Reappraisal	28.44 (6.15)	6	25	29	32	42	6	42	R3
ERQ Expressive Suppression	13.21 (5.07)	4	9	13	17	25	4	28	R3
Brief Self-Control Scale	44.25 (8.24)	20	39	45	50	65	13	65	R3
Intolerance of Uncertainty Scale	33.50 (8.50)	13	27	33	38.25	60	12	60	R3
UPPS-P negative urgency scale	7.89 (2.61)	4	6	8	9	16	4	16	R3
UPPS-P lack of premediation scale	12.90 (2.37)	5	12	13	15	16	4	16	R3
UPPS-P lack of perseverance scale	13.19 (2.11)	4	12	13	15	16	4	16	R3
UPPS-P sensation seeking scale	9.53 (2.89)	4	7	9	12	16	4	16	R3
UPPS-P positive urgency scale	8.58 (2.34)	4	7	9	10	16	4	16	R3

M (SD) = Mean and standard deviation of scores in dataset; Min = minimum score reported in dataset; 1^st^ Quartile = average responses within the first quartile of responses in dataset; Median = Median response in dataset; 3^rd^ Quartile = average responses within the third quartile of responses in dataset; Max = maximum score reported in dataset; Scale Min = minimum score possible on scale; Scale Max = maximum score possible on scale; Assessment = the assessment in which the scale/variable was included; Daily = assessment was part of daily survey, R1 = assessment was part of Round 1 one-time assessment, R2 = assessment was part of Round 2 one-time assessment, R3 = assessment was part of Round 3 one-time assessment; Worry Composite = sum of the five COVID-related worry questions in the daily survey; PHQ-9 (modified) = Patient Health Questionnaire-9 scale sans the question on suicidality (omitted due to IRB recommendation); GAD-7 = Generalized Anxiety Disorder-7 scale; ERQ = Emotion Regulation Questionnaire; UPPS-P = Urgency-Premeditation-Perseverance-Sensation Seeking-Positive Urgency (UPPS-P) Impulsive Behavior scale.

## References

[1] Cunningham, T., Fields, E., Garcia, S. & Kensinger, E. The influence of age on stress, worry, affect, and depression during the spring phase of the COVID-19 pandemic in the United States. *Emotion*. 10.1037/emo0000982 (2021). 10.1037/emo0000982 34138584

[2] Rodriguez-Seijas C (2020). Comparing the Impact of COVID-19-Related Social Distancing on Mood and Psychiatric Indicators in Sexual and Gender Minority (SGM) and Non-SGM Populations. Front Psychiatry..

[3] Breier A (1987). Controllable and uncontrollable stress in humans: alterations in mood and neuroendocrine and psychophysiological function. Am J Psychiat..

[4] Minkel JD (2012). Sleep deprivation and stressors: evidence for elevated negative affect in response to mild stressors when sleep deprived. Emotion..

[5] Haack M, Mullington JM (2005). Sustained sleep restriction reduces emotional and physical well-being. Pain..

[6] Berkman LF, Glass T, Brissette I, Seeman TE (2000). From social integration to health: Durkheim in the new millennium. Soc Sci Med..

[7] Berkman, L. F. & Glass, T. In *Social Epidemiology*. Vol 1 (ed. Berkman, L.F., Kawachi, I.) Ch. 7 (Oxford University Press, 2000).

[8] Cohen S, Wills TA (1985). Stress, social support, and the buffering hypothesis. Psychol Bull..

[9] Farioli-Vecchioli S, Sacchetti S, di Robilant NV, Cutuli D (2018). The role of physical exercise and omega-3 fatty acids in depressive illness in the elderly. Curr Neuropharmacol..

[10] Aguirre, L. E. & Villareal, D. T. in *Frailty: Pathophysiology, phenotype and patient care* Vol. 83 (ed. Fielding RA, Sieber C, Vellas B) Ch. 10 (Karger Publishers, 2015).

[11] Jahrami, H. *et al*. Sleep problems during COVID-19 pandemic by population: a systematic review and meta-analysis. *J Clin Sleep Med*, jcsm-8930. (2020). 10.5664/jcsm.8930 PMC7853219 33108269

[12] Tappe, A. US economy posts its worst drop on record. *CNN Business* https://www.cnn.com/2020/07/30/economy/us-economy-2020-second-quarter/index.html (2020).

[13] Harris PA (2009). Research electronic data capture (REDCap)—a metadata-driven methodology and workflow process for providing translational research informatics support. J Biomed Inform..

[14] Harris PA (2019). The REDCap consortium: Building an international community of software platform partners. J Biomed Inform.

[15] Watson, D. & Clark, L. A. The Panas-X. Manual for the positive and negative affect schedule-expanded form. *The University of Iowa’s Institutional Repository* https://ir.uiowa.edu/cgi/viewcontent.cgi?article=1011&context=psychology_pubs/ (1994).

[16] Kroenke K, Spitzer RL (2002). The PHQ-9: a new depression diagnostic and severity measure. Psychiat Ann..

[17] Buysse DJ, Reynolds CF, Monk TH, Berman SR, Kupfer DJ (1989). The Pittsburgh Sleep Quality Index: a new instrument for psychiatric practice and research. Psychiat Res..

[18] Ghotbi N (2020). The µMCTQ: An Ultra-Short Version of the Munich ChronoType Questionnaire. J Biol Rhythm..

[19] Spitzer RL, Kroenke K, Williams JB, Löwe B (2006). A brief measure for assessing generalized anxiety disorder: the GAD-7. Arch Intern Med..

[20] Garnefski N, Kraaij V (2006). Cognitive emotion regulation questionnaire–development of a short 18-item version (CERQ-short). Pers Indiv Differ..

[21] Heimberg RG (1999). Psychometric properties of the Liebowitz social anxiety scale. Psychol Med..

[22] Soto CJ, John OP (2017). Short and extra-short forms of the Big Five Inventory–2: The BFI-2-S and BFI-2-XS. J Res Pers..

[23] Morin CM, Belleville G, Bélanger L, Ivers H (2011). The Insomnia Severity Index: psychometric indicators to detect insomnia cases and evaluate treatment response. Sleep..

[24] Adan A, Almirall H (1991). Horne & Östberg Morningness-Eveningness Questionnaire: a reduced scale. Pers Indiv Differ.

[25] Cohen, S., Kamarck, T. & Mermelstein, R. in *Measuring stress: A guide for health and social scientists*. Vol. 1 (ed. Kessler, R.C., Cohen, S., Gordon, L.U.) Ch. 1, (Oxford University Press, 1994).

[26] Spreng RN, McKinnon MC, Mar RA, Levine B (2009). The Toronto Empathy Questionnaire: Scale development and initial validation of a factor-analytic solution to multiple empathy measures. J Pers Assess..

[27] Dugré JR, Giguére CÉ (2019). The Psychometric Properties of a Short UPPS-P Impulsive Behavior Scale Among Psychiatric Patients Evaluated in an Emergency Setting. Front Psychiatry.

[28] Carleton RN, Norton MPJ, Asmundson GJ (2007). Fearing the unknown: A short version of the Intolerance of Uncertainty Scale. J Anxiety Disord..

[29] Gross JJ, John OP (2003). Emotion regulation questionnaire. NeuroImage..

[30] Tangney JP, Baumeister RF, Boone AL (2004). High self‐control predicts good adjustment, less pathology, better grades, and interpersonal success. J Pers..

[31] Cunningham TJ, Fields EC, Kensinger EA (2020). Open Science Framework.

